# A Systematic Review and Meta-Analysis of Lipid Signatures in Post-traumatic Stress Disorder

**DOI:** 10.3389/fpsyt.2022.847310

**Published:** 2022-05-06

**Authors:** Veni Bharti, Aseem Bhardwaj, David A. Elias, Arron W. S. Metcalfe, Jong Sung Kim

**Affiliations:** ^1^Department of Community Health and Epidemiology, Faculty of Medicine, Dalhousie University, Halifax, NS, Canada; ^2^Health and Environments Research Centre (HERC) Laboratory, Faculty of Medicine, Dalhousie University, Halifax, NS, Canada; ^3^Canadian Health Solutions Inc., Saint John, NB, Canada; ^4^Dalhousie Medicine New Brunswick, Dalhousie University, Halifax, NS, Canada; ^5^Canadian Imaging Research Centre, Saint John, NB, Canada

**Keywords:** post-traumatic stress disorder, psychiatric disorders, major depressive disorder, lipids, fatty acids

## Abstract

**Background:**

Research assessing lipid levels in individuals diagnosed with post-traumatic stress disorder (PTSD) has yielded mixed results. This study aimed to employ meta-analytic techniques to characterize the relationship between the levels of lipid profiles and PTSD.

**Methods:**

We performed meta-analyses of studies comparing profiles and levels of lipids between PTSD patients and healthy individuals by searching Embase, Ovid Medline, Scopus, PsycINFO, and Cochrane databases for the studies until March 2021. Meta-analyses were performed using random-effects models with the restricted maximum-likelihood estimator to synthesize the effect size assessed by standardized mean difference (SMD) across studies.

**Findings:**

A total of 8,657 abstracts were identified, and 17 studies were included. Levels of total cholesterol (TC) (SMD **=** 0.57 95% CI, 0.27–0.87, *p* = 0.003), low-density lipoprotein (LDL) (SMD = 0.48, 95% CI, 0.19–0.76, *p* = 0.004), and triglyceride (TG) (SMD = 0.46, 95% CI, 0.22–0.70, *p* = 0.001) were found to be higher, while levels of high-density lipoprotein (HDL) (SMD = –0.47, –0.88 to –0.07, *p* = 0.026) were found to be lower in PTSD patients compared to healthy controls. Subgroup analysis showed that TG levels were higher in PTSD patients who were on or off of psychotropic medications, both < 40 and ≥ 40 years of age, and having body mass index of < 30 and ≥ 30 compared to healthy controls.

**Interpretation:**

This work suggested dysregulation of lipids in PTSD that may serve as biomarker to predict the risk. The study will be useful for physicians considering lipid profiles in PTSD patients to reduce cardiovascular morbidity and mortality.

## Introduction

Post-traumatic stress disorder (PTSD) is a mental health condition that appears after exposure to a life-threatening or traumatic event or repeated exposure to traumatic events (1). PTSD is characterized by nightmares and flashbacks of previous traumatic events, sleep disturbance, disturbing thoughts, and avoidance of reminders of trauma (2). It has also been found that those with PTSD have an increased risk of cardiovascular disorder related morbidity (3, 4) and mortality (5). A considerable body of evidence suggests that patients with PTSD often exhibit dysregulated lipid metabolomic profiles (6–20).

When considering the association between the profiles and levels of lipid biomarkers and PTSD, it is worth noting that the human brain comprises 50–60% lipid constituents of its dry weight (21). Almost 80% of the adult brain’s cholesterol is present in myelin forming oligodendrocytes (22). Myelin is important for increasing the speed of electrical impulses along the nerve fiber (23), protection of neurons by insulation (23), protection from oxidative stress (24) and maintaining integrity of the blood brain barrier (25). Lipids play important roles in the brain, including neurogenesis, synaptogenesis, myelin formation, and impulse conduction (26). Availability of cholesterol is one of the limiting factors in synaptogenesis and is vital for continuous synaptogenesis. It is also important for stability of neurotransmitters (27). Both pre-and post-synaptic areas are rich in cholesterol and organize the synaptic proteins. They are essential in maintaining general neurotransmission process (28). Fatty acids, polyunsaturated fatty acids (PUFA) in particular, act as “fuel partitioners” by downregulating the genes for lipid synthesis while simultaneously upregulating the genes involved in fatty acid oxidation (29). PUFA modulates the electrical current in neurons by regulating ion channel (30, 31). PUFA can also alter the transcription of genes involved in lipid synthesis (32). Pathophysiology of PTSD entails synaptic loss (33), increased myelination (34), and white matter abnormalities (35) and reduction in cortical thickness (36) in the brain suggesting the role of lipids in the pathogenesis of PTSD. Thus, any changes in lipid levels may affect mood and altered lipid patterns may serve as biomarkers for early diagnosis of mood disorders.

Despite the clear theoretical and mechanistic underpinnings, the observational and epidemiological studies reporting the relationship between serum lipid levels and PTSD are controversial. Some studies have shown that PTSD patients have lower total cholesterol (TC) levels (5), high- density lipoprotein (HDL) levels (5, 7, 10–13, 16), low-density lipoprotein (LDL) levels (17) and triglyceride (TG) levels (15); others have reported higher levels of TC (6, 11–14, 17, 19, 37), HDL (9, 15), LDL (6, 8, 9, 11–14, 16), and TG (6, 9, 11–14, 18, 19). Given the inconsistency in the literature and as of yet, the absence of a meta-analysis comparing the lipid profiles and levels between PTSD patients and healthy controls, evidence synthesis in a meta-analysis may be of use. Therefore, the primary aim of this meta-analysis was to assess the profiles and levels of lipid biomarkers reported in studies of individuals with PTSD and discuss the potential roles of lipid and fatty acid parameters in PTSD. Previous studies suggest a direct association between dysregulated lipid and lipoprotein levels with the cardiovascular events (38, 39), therefore making it important to comprehensively synthesize the current evidence examining lipids and fatty acids compared between PTSD patients and healthy individuals. This is the first meta-analysis comparing the profiles and levels of lipid and fatty acid biomarkers as a continuous measure between PTSD patients and healthy controls and will be useful for clinicians in assessing the risk of cardiovascular events in PTSD patients.

## Methods

### Search Strategy and Selection Criteria

We followed the Preferred Reporting Items for Systematic Reviews and Meta-analyses (PRISMA) guidelines to perform this meta-analysis (40). Five databases including Embase, Ovid Medline, Scopus, PsycINFO, and Cochrane were searched for articles published up to March 2021 using the following keywords: “cholesterol” OR “LDL” OR “HDL” OR “VLDL” OR “triglyceride” OR “apolipoprotein A” OR “apolipoprotein B” OR “docosahexanoic acid” OR “omega 3 fatty acid” OR “omega 6 fatty acid” OR “polyunsaturated acid” OR “triglyceride” AND “post-traumatic stress disorder” OR “PTSD.” A full search strategy is appended in Supplementary Information. Emtree terms were used in Embase database. The search was limited to original research articles and human studies.

Search lists from all databases were imported to Zotero software (2012, United States) and duplications were removed. All studies were imported to Covidence software (2014, United States) for title and abstract screening and full-text screening by two reviewers, VB and AB independently. Any conflicts at either step were resolved by JSK. The gray literature and articles published in any other language than English were excluded manually by reviewers independently.

The original articles measuring the levels of lipids and fatty acids in blood, serum, and plasma of PTSD patients compared with healthy controls were included. Only the studies that diagnosed PTSD patients using any edition of the Diagnostic and Statistical Manual of Mental Disorders and International Classification of Disease (ICD-10) codes were included. Healthy controls were defined as subjects without having any medical conditions and not using any substance of abuse and antihyperlipidemic medication. Studies having PTSD patients of all age groups were included. Studies having any comorbid cardiovascular disorder (CVD), metabolic disorder, and psychiatric disorders were excluded. PTSD induced by any CVD were not included because CVD itself can increase the level of lipids and may introduce bias in the present study. Studies not representing the continuous measure of lipid parameters were also excluded.

### Data Extraction

Data was extracted manually on Excel sheets by both reviewers VB and AB. The characteristics extracted from each study were the name of the first author, publication year, mean age and standard deviation (SD), number of PTSD patients and control group, mean and SD of TC, LDL, HDL, very-low density lipoprotein (VLDL), TG, BMI, smoking, alcohol intake and antipsychotic and antihyperlipidemic medication status of PTSD group in all studies. The mean values were reported in International System of Units (SI) units.

### Measurement of Inter-Rater Reliability

Measurement of inter-rater reliability was measured by calculating cohen’s kappa coefficient using Covidence software. Kappa coefficient was calculated based on proportionate agreement between two reviewers, random agreement probability, probability of yes/no to select articles.

### Quality Assessment

Quality assessment of the studies was performed according to New Castle Ottawa (NOS) scale.

### Data Analysis

Meta and dmetar packages of R Studio statistical software (4.0.0) were used to conduct this meta-analysis. The meta-analysis was performed when two or more studies reported the mean and SD values of lipids in PTSD and healthy control groups (41). The random-effects model with the restricted maximum-likelihood estimator was used to synthesize the effect size across the studies. This model incorporates both within-study and between-study variability (42). Standardized mean difference (SMD) was used to assess the effect size due to the variability of assessment methods used by different studies. SMD was calculated using Cohen’s D (42). Cohen’s D is an effect size used to indicate the standardized difference between two means. The significance level for this meta-analysis model was a *p*-value below 0.05. Effect sizes 0.2, 0.5, and 0.8 were considered as low, moderate, and high effect, respectively (42). Upper and lower limits of 95% confidence intervals (CI) for Cohen’s effect size were also calculated. Heterogeneity across studies was assessed by Q statistics while inconsistency across studies was assessed by *I*^2^-value (43). *I*^2^-value of below 25% was considered as low heterogeneity, 25–75% was considered as moderate, and > 75% was considered as high heterogeneity (43). The total amount of heterogeneity was estimated by heterogeneity variance (τ^2^) (43). Publication bias was assessed by Egger’s linear regression test (44). This test is used to calculate the asymmetry of the funnel plot. A *p* < 0.05 was considered to show significant asymmetry and publication bias (45). This test was not performed when studies less than 10 were present and represented diagrammatically with funnel plots as recommended by Cochrane Collaboration (43).

Second, we performed subgroup analysis of lipids to determine the effect of age, sex, geography, BMI, and use of antipsychotic medications on lipid levels in PTSD patients. Sub-group analysis was not performed where studies below 10 were presented to avoid the reduction of the power of test (41). We used age as a variable for subgroup analysis because LDL levels rises with age due to reduced hepatic LDL receptor expression, which in turn reduces the capacity for removal of LDL (46). We specifically chose the age groups under and above 40 years because of the previous literature suggesting an increase in LDL concentration after 40 years of age (47). Similarly, sex was chosen as a variable because previous studies have reported that males and premenopausal females have different blood lipid profiles. Men in general have higher LDL, TG, and VLDL concentrations than pre-menopause women (48, 49). We stratified the studies as males only, females only, and both sex studies because some of the studies had almost equal ratio of male and female participants. Studies having both male and female participants were not included in the male and female specific classes because that may interfere with the analysis and may introduce bias when investigating the influence of sex on lipid levels. Genetics and environmental factors also shape the lipid profiles (50) of an individual and that may introduce the difference between lipid profiles in people living in different geographical locations. Obesity is also a risk factor for dyslipidemia and approximately 60–70% people having high BMI are known to have high levels of VLDL, TG, and non-HDL cholesterols (51–54). To understand the potential effect of obesity on lipid levels, we stratified the BMI levels above and below 30 because BMI at and above 30 is considered as obesity (55). We also considered antipsychotic medications as a variable for the subgroup analysis because these medications are reported to increase the TG levels (56) and decrease HDL levels (57).

## Results

### Study Selection

**FIGURE 1:**
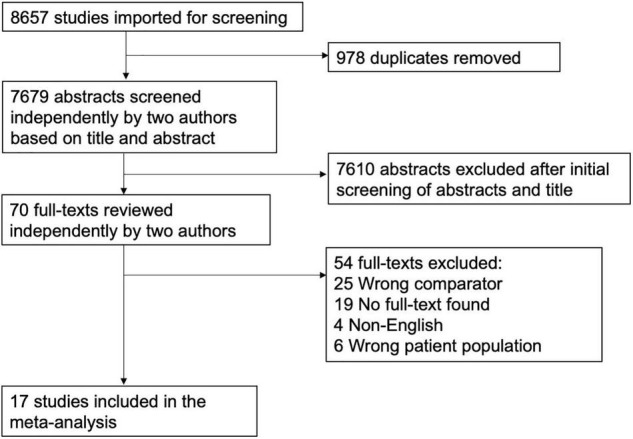
Flow Diagram of the literature search and study selection. The flow diagram represents the number and reasons for elimination of studies at every step based on the selection criteria.

Of the 8,657 citations retrieved, we included 17 studies for final data inclusion to compare the levels and profiles of lipids between PTSD patients and healthy controls. The inclusion-exclusion criteria for final study selection are shown in Figure 1. We excluded studies that did not provide lipid levels as continuous measures and studies that included any cardiovascular event as the stressor that induced the PTSD condition and studies that included subjects with other psychiatric disorders comorbid with PTSD. The characteristics of the included studies including author name and year of publication, country, lipid markers studied, number of PTSD patients and healthy controls, mean age and SD of PTSD patients and healthy controls, and medication status are described in Table 1. 40% of the selected studies showed combat-related activities as the stressor that induced PTSD. Measurement of Cohen’s kappa for measuring inter-rater reliability measurement is shown in Table 2. Quality assessment of the studies was performed according to Newcastle Ottawa Scale and are presented in Table 3.

**TABLE 1:** Characteristics of studies included in the meta-analysis.

Sr. no.	References	Marker	N (PTSD, HC)	Age (PTSD, HC)	Medicated
1	Talbot et al. (6)	TC, LDL, TG, VLDL	94 (44, 50)	30.55 ± 6.57, 30.34 ± 8.11	No
2	Tae et al. (7)	HDL	107 (68, 39)	45.18 ± 8.05, 42.72 ± 10.0	No
3	Jergović et al. (8)	LDL	101 (69, 32)	47.12 ± 5.92, 45.56 ± 7.24	Yes
4	Dedert et al. (9)	HDL, LDL, TG	134 (63, 71)	41.3 ± 11.6, 39.6 ± 14	No
5	Dennis et al. (10)	HDL	220 (103, 117)	30.49 ± 5.48, 27.9 ± 5.52	No
6	Karlovic et al. (11)	TC, HDL, LDL, TG	82 (43, 39)	41.3 ± 8.3, 43.8 ± 10.1	Yes
7	Karlovic et al. (12)	TC, HDL, LDL, TG	102 (53, 49)	34 ± 5.4, 35 ± 4.3	Yes
8	Kulenović et al. (13)	TC, HDL, LDL, TG, VLDL	100 (50, 50)	45, 45	No
9	Maia et al. (14)	TC, LDL, TG	101 (11, 90)	35.73 ± 7.12, 33.11 ± 5.38	NA
10	Lihua et al. (15)	HDL, TG	2,867 (201, 2,666)	25, 35	NA
11	Hamazaki et al. (16)	HDL, LDL	30 (15, 15)	47 ± 16, 36 ± 15	NA
12	de Vries et al. (17)	TC, LDL	94 (49, 45)	46 ± 10.4, 46.6 ± 10	Yes
13	Linnville et al. (18)	TG	154 (89, 65)	61 ± 6, 61 ± 6	NA
14	Ahmadi et al. (5)	TC, HDL	637 (88, 549)	59 ± 9, 58 ± 10	NA
15	Su et al. (19)	TC, TG	709 (75, 634)	17 ± 0.61, 16.9 ± 0.58	NA
16	Ansari and Ahmed (37)	TC	65 (50, 15)	35.1 ± 1.6, 35.1 ± 1.6	NA
17	McLeay et al. (95)	TG	214 (108, 106)	68.5 ± 4.1, 69.2 ± 4.2	NA

*PTSD, post-traumatic stress disorder; HC, healthy control; TC, total cholesterol; LDL, low density-lipoprotein; HDL, High density lipoprotein; TG, triglyceride; VLDL, very low density lipoprotein; NA, not available.*

**TABLE 2:** Measurement of inter-rater reliability.

Measurement of inter-rater reliability at title and abstract level		
	**Reviewer 1**	
**Reviewer 2**	**Yes**	**No**
Yes	66	19
No	35	7,559
Proportionate agreement: 0.99296		
Probability of yes: 0.00014		
Probability of no: 0.97592		
Random agreement: 0.97606		
Cohen’s kappa coefficient: 0.70		

**Measurement of inter-rater reliability at full text selection level**		

	**Reviewer 1**	
**Reviewer 2**	**Yes**	**No**

Yes	15	4
No	4	47
Proportionate agreement: 0.88571		
Probability of yes:0.0736		
Probability of no: 0.53081		
Random agreement: 0.60448		
Cohen’s kappa coefficient: 0.71		

**TABLE 3:** Quality assessment of studies.

Sr. No.	References	Study type	Selection	Comparability	Outcome	Total
1	Talbot et al. (6)	Cross-sectional	4	1	3	8
2	Tae et al. (7)	Prospective cohort	3	1	3	7
3	Jergović et al. (8)	Case control	4	1	2	7
4	Dedret et al. (9)	Cross-sectional	4	1	3	8
5	Dennis et al. (10)	Cross-sectional	4	1	3	8
6	Karlovic et al. (11))	Cross-sectional	3	1	3	7
7	Karlovic et al. (12)	Cross-sectional	3	1	3	7
8	Kulenović et al. (13)	Cross-sectional	3	1	3	7
9	Maia et al. (14)	Cross-sectional	3	1	3	7
10	Lihua et al. (15)	Cross-sectional	4	1	3	8
11	Hamazaki et al. (16)	Cohort	4	1	3	8
12	de Vries et al. (17)	Cross-sectional	4	1	3	8
13	Linnville et al. (18)	Cross-sectional	3	1	3	7
14	Ahmadi et al. (5)	Cross-sectional	4	1	3	8
15	Su et al. (19)	Cross-sectional	5	1	3	9
16	Ansari and Ahmed (37)	Cohort	3	1	2	6
17	McLeay et al. (95)	Cross-sectional	5	1	3	9

### Meta-Analysis of Lipid Levels in Post-traumatic Stress Disorder

**FIGURE 2:**
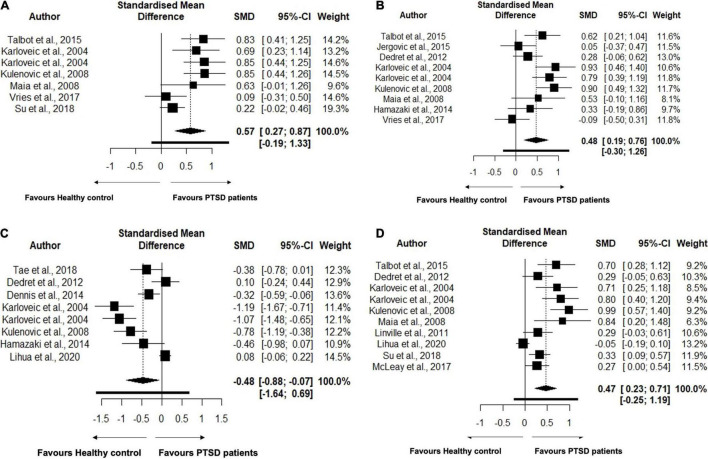
**(A)** Forest plot showing lipid parameter Total Cholesterol (TC) levels in the patients with Post-traumatic Stress Disorder (PTSD) and healthy controls. The black squares correspond to the standardized mean difference (SMD) of each study and horizontal bars represent 95% Cl of each study. **(B)** Forest plot showing lipid parameter Low Density-Lipoprotein (LDL) levels in the patients with Post-traumatic Stress Disorder (PTSD) and healthy controls. The black squares correspond to the standardized mean difference (SMD) of each study and horizontal bars represent 95% Cl of each study. **(C)** Forest plot showing lipid parameter High Density-Lipoprotein (HDL) levels in the patients with Post-traumatic Stress Disorder (PTSD) and healthy controls. The black squares correspond to the standardized mean difference (SMD) of each study and horizontal bars represent 95% Cl of each study. **(D)** Forest plot showing lipid parameter Triglyceride (TG) levels in the patients with Post-traumatic Stress Disorder (PTSD) and healthy controls. The black squares correspond to the standardized mean difference (SMD) of each study and horizontal bars represent 95% Cl of each study.

TC levels were analyzed using data from 9 studies, comprising 463 PTSD patients and 887 healthy controls in Table 4. No changes in the TC levels were found between PTSD patients and healthy controls (SMD = 1.51, 95% CI, –0.94 to 3.96, *p* = 0.194) in the original analysis. We also performed sensitivity analysis to reassess the robustness of the findings for TC in primary meta-analysis (58). TC levels were significantly increased in the PTSD patients compared to controls (SMD = 0.46, 95% CI, 0.12–0.81, *p* = 0.014) (Figure 2A) after removing an outlier study (37). Heterogeneity was found to be high between samples, with *I*^2^ 83.6%. Closely looking at each study, we found that the study (5) included the TC levels data from medical records rather than testing it at the time of recruitment. Since testing for TC levels may be performed at different frequencies for each individual, this may lead to bias in the data and may shift the values in each group. Second, this study also did not report if lipid levels were measured in serum or plasma. A study has shown that the choice of anticoagulant while making the sample may affect the measurements of lipid profiles (59). As a result, we reassessed our analysis strategy, determining the effect size by excluding this study. After excluding this study, we found that TC levels remained significant with a larger effect size (SMD = 0.57, 95% CI, 0.27–0.87, *p* = 0.003) (Figure 2A). This reduced the heterogeneity between studies, with *I*^2^ 66.4%. We found 9 studies comprising 397 PTSD patients and 441 healthy controls to compare the levels of LDL between PTSD and healthy controls. PTSD patients exhibited significantly high levels of LDL compared to healthy controls (SMD = 0.48, 95% CI, 0.19–0.76, *p* = 0.004) (Figure 2B). No changes in the results were observed when we performed the sensitivity analysis for LDL. We did not find any changes in the HDL levels between PTSD patients and healthy controls (SMD = –0.32, 95% CI, –0.83 to 0.19, *p* = 0.187). There was high heterogeneity between samples, with *I*^2^ 94.4%. When the Ahmadi et al. (5) study was removed as an outlier, HDL levels were found to be significantly lower in PTSD patients than healthy controls (SMD = –0.48, 95% CI, –0.88 to –0.07, *p* = 0.026); heterogeneity between studies was still high with *I*^2^ 88.5% (Figure 2C). TG levels were analyzed using data from 10 studies, comprising 737 PTSD patients and 3,820 healthy controls. TG levels were significantly increased in PTSD patients compared to healthy controls (SMD = 0.46, 95% CI, 0.22–0.70, *p* = 0.002) (Figure 2D). There was significant between sample heterogeneity with *I*^2^ 81.1%. Egger’s test revealed asymmetry of the funnel plot and indicates publication bias with Z-statistic value 4.864 (*p* = 0.0001). After removing the outlier study Lihua et al. (15), TG levels remained significantly high in PTSD patients compared to healthy controls, but heterogeneity among studies was reduced to moderate levels with *I*^2^ 55.5%. We did not find any difference in the VLDL between PTSD patients and healthy controls (SMD = 0.83, 95% CI, –0.60 to 2.25, *p* = 0.085). Although in our search strategy we exclusively used the keywords to search for articles comparing apolipoproteins, polyunsaturated fatty acids, and monounsaturated fatty acids, we were not able to find at least two publications measuring these parameters as continuous measures comparing between PTSD patients and healthy controls. Hence, due to the limited availability of the publications, we were not able to perform meta-analysis for these parameters.

**TABLE 4:** Meta-analysis of lipid levels in PTSD.

Lipid/fatty acid/lipid regulatory protein	Q statistic (DF; *p*-value)	SMD (95%CI)	*p*-value	*I*^2^ (%)	τ^2^	Egger’s test Z-statistic (*p*-value)
TC	17.86 (6; 0.006)	0.57 (0.27–0.87)	0.003	66.4	0.07	NA
LDL	23.48 (8; 0.002)	0.48 (0.19–0.76)	0.004	65.9	0.09	NA
HDL	60.79 (7; < 0.0001)	–0.48 (–0.88 to –0.07)	0.026	88.5	0.69	NA
TG	47.67 (9; < 0.0001)	0.46 (0.22–0.70)	0.001	81.1	0.08	4.864 (*p* = 0.0001)
VLDL	0.56 (1; 0.4538)	0.83 (–0.60 to 2.25)	0.085	0	0	NA

*TC, total cholesterol; LDL, low density-lipoprotein; HDL, High density lipoprotein; TG, triglyceride; VLDL, very low density-lipoprotein; NA, not available. Egger’s test was not performed for parameters having < 10 studies.*

### Subgroup Analyses

#### Triglyceride

To better understand the results of the meta-analysis, relevant *a priori* subgroups were investigated (Table 5). Subgroup analysis was performed to analyze the effect of sex, age, geography, use of psychotropic medication, and geographical location on TG levels. Differences in TG concentration levels between PTSD patients and healthy controls were present in the studies performed in males (*p* = 0.0001) but not in females (*p* = 0.097). Similarly, studies involving both sexes with approximately the same ratio also did not show a difference in TG levels (*p* = 0.169). Interestingly, TG levels were found to be higher in PTSD patients in both below 40 years of age (*p* < 0.0001) and 40 years and above (*p* = 0.006). PTSD patients exhibited higher levels of TG levels in the studies performed in both America (*p* < 0.0001), Europe (*p* ≤ 0.0001), and Australia (*p* = 0.05) but not in the studies performed in Asia (*p* = 0.493). TG levels were higher in the PTSD patients who were on (*p* = 0.001) or off (*p* = 0.001) on antipsychotic medications and both BMI over 30 (*p* < 0.0001) and BMI less 30 (*p* = 0.0004).

**TABLE 5:** Subgroup analyses for studies included in the analysis that assessed the triglyceride (TG) levels in PTSD patients vs. Healthy Controls.

Subgroup	No. of studies	SMD (95% CI)	*p*-value	*I* ^2^
**Sex**				
Males	6	0.57 (0.27–0.86)	0.0001	0.88
Females	1	0.28 (–0.05 to 0.62)	0.097	NA
Both sexes	3	0.29 (–0.12 to 0.70)	0.169	0.87
**Age**				
< 40 years	5	0.48 (0.19–0.77)	< 0.0001	0.64
≥ 40 years	5	0.45 (0.12–0.78)	0.006	0.87
**Geography**				
America	4	0.45 (0.20–0.70)	0.0004	0.35
Europe	3	0.84 (0.68–0.99)	<0.0001	0
Asia	2	0.12 (–0.23 to 0.49)	0.930	0.85
Australia	1	0.26 (0.003–0.53)	0.05	0
**Use of psychotropic drugs**				
Psychotropic medications	2	0.55 (0.22–0.89)	0.001	0.66
No psychotropic medications	3	0.64 (0.24–1.04)	0.001	0.71
**BMI**				
≥30	2	0.27 (0.25–0.29)	<0.0001	0
<30	7	0.51 (0.22–0.79)	0.0004	0.86

## Discussion

We sought to determine the association between the profiles of lipids and fatty acids and having a diagnosis of PTSD using the first meta-analysis study by comparing a slate of lipid and fatty acid measurements between PTSD and healthy control samples. At first, no changes in the TC levels were observed. However, the levels of TC were found to be significantly high in PTSD patients compared to controls after exclusion of the outlier study (37). While the effect size calculated by SMD ranged from –0.18 to 0.85, this study showed SMD value of 10.36, increasing TC values in PTSD patients at extremely high levels. This may be due to differences in the selection of participant characteristics compared to other studies we included in the analysis of effect size. This study did not select any cutoff values for BMI or measure waist circumference while doing participant selection. Previous studies have found that high BMI and increased body weight are risk factors for dyslipidemia (51–53). Therefore, the lack of control of these variables may have been a significant contributing factor to the unexpected findings in this study. Levels of LDL and TG were found to be higher in PTSD patients than healthy controls. At first, no changes in HDL levels were observed between PTSD patients and healthy controls, but after exclusion of an outlier study by Ahmadi et al. (5), HDL was found to be significantly reduced in PTSD patients compared to healthy controls. It is important to note that this particular study reported the HDL levels from medical records. Due to interindividual differences of lipid testing being done, this may introduce bias in the analysis. We only included studies that measured lipid levels at the time of recruitment. One possibility here relates to previous findings that patients having PTSD show elevated sympathetic nervous system (SNS) activity (60). Activation of the SNS leads to enhanced leptin release, thereby surging lipolysis and lipid mobilization from white adipose tissue. Increased lipolysis leads to augmented release of free fatty acids that are further transported to the liver and increase the TG production. A previous study has reported that sympathetic denervation in the liver decreased the TG-VLDL secretion (61), suggesting that SNS directly activates the liver lipogenesis. Fatty acid release is increased by stimulating hepatic VLDL-TG production and increased lipolysis in white adipose tissue. Due to the lipolysis of in white adipose tissue, free fatty acids are released that are further transported to the liver to increase the production of TG (62). Previous studies have shown that PTSD patients exhibit high levels of the stress hormone cortisol even in recovery phase (63, 64). Cortisol when present in excess, also increases lipolysis and elevates the free fatty acid levels that further increases VLDL secretion and TG storage in the liver (65). Increased free fatty acid levels and cortisol also enhance the activity of HMG-CoA reductase enzyme activity in the liver, stimulating the synthesis of total cholesterol (65). In the presence of high cortisol, LDL-receptor activity is also reduced, leading to high levels of LDL (66, 67). Supporting evidence for this interpretation was found here in the subgroup analysis of BMI where elevated TG levels were present even in lower BMI PTSD samples compared to lower BMI healthy control samples, discussed in more detail in the following section.

Our subgroup analysis for TG showed higher levels of TG in PTSD patients in male-specific studies when compared to healthy controls. No changes were observed in TG levels in female-specific studies and other studies had almost equal ratios of males and females. Our observation is in agreement with a previous study which showed males had higher TG levels than females (68). One of the possible explanations of having higher TG levels in men may be due to the high waist/hip ratio in men compared to women. High waist/hip ratio is an indicator of visceral fat and could be a major determinant of hepatic exposure to free fatty acids released by portal adipose tissues (69). However, due to the scarcity of data on waist/hip ratio, we were not able to analyze this data. Another study reported that the proportion of men who were 45 or over 45 years of age

having low testosterone levels had an increase in TG/HDL ratio quartile (70). Low testosterone levels are associated with high deposition of visceral fat, central obesity, and other metabolic syndromes including insulin resistance (71, 72). When we closely looked at our analysis, 4 out of 6 male-specific studies were reported in males over 40 years, suggesting a possibility of reduced testosterone that may increase TG levels and reduce HDL levels. Previous studies have suggested that the luteal phase in females is shown to have reduced TG levels than follicular phase (73). Studies analyzed in the present work did not report in which phase of the menstrual cycle the sample was taken from female participants. Our study also indicated higher TG levels in PTSD patients than healthy controls in both groups with BMI below 30 and 30 and above. BMI is a measure to determine the weight range of the body by calculating body weight and height. This parameter classifies individuals as underweight, normal, overweight, or obese. However, one of the limitations of this scale is that it does not demonstrate how much fat and muscle one has in the body. Measurement of waist circumference is another tool that can be used to determine central obesity and can also be used to determine the waist/hip ratio. Indeed, it has been shown that waist circumference is positively correlated with TG levels (74). Although we did not perform subgroup analysis of TC, LDL, and HDL to investigate the effects of BMI on these parameters in PTSD patients due to the presence of less than 10 studies, our raw data suggests increased TC and LDL, and decreased HDL in PTSD patients with BMI of 30. Increased central obesity and visceral tissues are known to be correlated with high TC and LDL levels, and reduced HDL levels (74). People with high waist circumference have high TG accumulated in the fat tissues that may lead to increased conversion of TG-rich LDL to small, dense LDL with longer half-life by hepatic lipase enzymes. When TG levels are high, HDL particles are enriched with TG that can be catabolized faster than cholesteryl esters containing HDL particles, thereby reducing the levels of HDL upon increase in TG levels (75).

Our study also showed higher TG levels in the PTSD patients than controls in American and European studies than the studies performed in Asia. This may be explained by the fact reported in a previous study which reported that Asians exhibit more somatic symptoms such as reduced appetite when depressed than non-Asians (76). Since PTSD patients represent depressed mood, lesser food intake in these patients may lead to reduced TG in Asian studies. However, the studies included in the meta-analysis did not report dietary intake. Another possible reason for higher TG in non-Asian studies than Asian studies may be due to the presence of a low number of Asian studies. Out of 10 studies representing a comparison of TG levels between PTSD patients and healthy controls, only 2 studies were performed in Asia. The low number of Asian studies may be due to the lower risk of Asians for the development of PTSD upon exposure to the traumatic events (77).

Our study also reported higher TG levels in the PTSD patients who were both on or off of antipsychotic medications, when compared to healthy controls. One of the previous studies has suggested that the patients having psychiatric disorders present metabolic dyslipidemia along with features of metabolic syndrome and other risk factors for cardiovascular disorder (78). Antipsychotic medications including but not limited to olanzapine, quetiapine, clozapine, amitriptyline, are reported to increase metabolic disturbances including high TG levels (78, 79). Previous studies reported that these medications elevate lipid levels by stimulating the sterol regulatory element binding protein that increases gene expression related to lipid biosynthesis (80, 81). In our study, 20% of the studies reported that their participants were on antipsychotics, while 30% reported no use of antipsychotics by PTSD patients. The other 50% of the studies did not report data on the current use of antipsychotics by PTSD patients. Interestingly, two studies that reported no use of antipsychotics and higher TG levels in PTSD patients showed that these patients were current smokers (11, 12) and one of these two studies reported the use of alcohol by these patients.^9^ This observation is consistent with previous studies suggesting that smoking lead to high serum TG levels (82, 83). Alcohol intake also increases the synthesis of VLDL particles in the liver, which serves as the main source of TG production; thereby increasing the level of TG (84).

TG levels were found to be higher in the PTSD patients both in below 40 and 40 and above years of age than when compared to healthy controls. In older age, fat accumulation starts increasing that may lead to increases in TG levels. Diet, alcohol intake, lifestyle, smoking, central obesity, and fat content all contribute to increases in TG levels. However, our reviewed studies did not report data on these parameters, limiting our analysis for finding the reasons behind increased TG levels in younger age counterparts.

The mechanism for lipid dysregulation and its effects on swinging mood in PTSD is not well understood. It has been proposed that cholesterol present in the form of “lipid rafts” in the synaptosomal membrane might determine the number of serotonin receptors (85) and dopamine transporters (51, 86), thus making an impact on neurotransmitter signaling. Activation of these receptors are important for the regulation of mood (87). Patients having PTSD have shown decreased concentrations of serotonin in the dorsal and median raphe nuclei and increased dopamine levels (88). The dysregulation of these neurotransmitters interferes with the fear conditioning circuits by disturbing the dynamics between the amygdala and hippocampus and by affecting the mesolimbic system (88). Fear conditioning plays a major role in PTSD. In PTSD, fear learned in a traumatic situation can be triggered by a variety of provocations that resemble the initial trauma (89). Thus, dysregulated lipids may affect neurotransmitter signaling, further affecting mood and other behavioral symptoms seen in PTSD patients such as having flashbacks of traumatic events and having low mood.

Important strengths of our systematic review include searching articles from many databases, reducing the chances of losing articles published on lipid levels in PTSD patients. We also included all types of study designs for this meta-analysis such as prospective studies and cross-sectional studies. Another important strength to be considered is that we strictly included articles comparing lipid levels between PTSD patients and healthy controls only. We excluded other comorbid psychiatric disorders because the hypothalamic-pituitary axis is dysregulated in other psychiatric disorders as well, which would lead to inappropriate measurement of dysregulation of lipids related to this particular psychiatric condition.

However, several limitations in the present study should be acknowledged. We had less than 10 studies for TC, LDL, HDL, and VLDL to investigate the effects of sex, age, BMI, antipsychotic medications, and geographical location on these lipids. Most of our studies reported BMI as a measure of obesity. We were not able to identify the role of visceral fats on the levels of these lipids that play an important role in the dysregulation of lipids. This may be due to the presence of more cells per unit mass in intra-abdominal tissue, more glucocorticoid receptors, and greater catecholamine induced lipolysis compared to subcutaneous adipose tissue (74). Most of our studies did not include dietary habits and other lifestyle parameters and limited our analysis. In our search strategy, we included PUFA and MUFA to compare the data of these fatty acids between PTSD patients and healthy controls. However, due to the presence of less than 2 studies, we were not able to accomplish that goal. Another limitation of our study is that our protocol was not pre-registered for this review. Limited number of published studies did not allow us to explore variations due to various factors that may affect the analysis such as sample characteristics or diagnostic method to assess lipid levels. Most of the studies we included had PTSD cases due to war or natural disaster, but further research is needed to examine the risk of dyslipidemia in PTSD patients who were exposed to other types of traumas, i.e., sexual abuse, violence etc. We were not able to find any published literature reporting the levels of lipids in PTSD patients due to childhood stress or in children having PTSD.

In summary, PTSD is positively associated with the levels of TC, LDL, and TG and negatively associated with the levels of HDL, indicating that this pattern may serve as a biomarker to assess the risk of PTSD in potential patients that otherwise have no obvious health-related indicators for exhibiting this lipid profile. In our previous meta-analysis study (90), pattern of the lipid profiles of patients with major depressive disorder (MDD) were found to be different than our current analysis in PTSD patients. MDD patients showed a reduction in TC levels, increase in the levels of TG and VLDL, and no changes in LDL and HDL levels (90). However, our results indicate that PTSD patients show an increase in total cholesterol, LDL, and TG, and a reduction in HDL levels. MDD and PTSD both share certain symptoms such as anxiety, recurrent suicidal ideations, negative feelings, and social impairments that make their differential diagnosis difficult. A study published in 2018 suggested that PTSD patients represent high dehydroepiandrosterone (DHEA) levels in contrast to MDD patients who exhibit low DHEA levels (91). Previous studies have shown that long-term oral DHEA therapy reduced HDL levels (92, 93), which is consistent with our results showing reduced HDL in PTSD patients and not in MDD patients (90). HDL plays a key role in reverse cholesterol transport and promotes the efflux of cholesterol from peripheral tissues, hepatocytes, and macrophages, leading to an overall reduction in the levels of TC (94). Since MDD patients represented no changes in HDL levels compared to healthy controls (90), this may explain decrease in TC in contrast to PTSD patients who show increased TC. Second, this work will be useful for physicians when considering the risk while assessing lipid levels in PTSD patients to reduce the cardiovascular morbidity and mortality in these patients. More mechanistic studies are required to understand the association between lipid levels and PTSD. Since the number of studies to understand the source of heterogeneity was small for TC, LDL, and HDL levels, more longitudinal studies are required to understand the levels of these parameters in PTSD patients. Further studies are required to study the predictive values of lipid levels to assess the risk of cardiovascular mortality and morbidity in PTSD.

## Data Availability Statement

The original contributions presented in the study are included in the article/Supplementary Material, further inquiries can be directed to the corresponding author/s.

## Author Contributions

VB and AB selected the abstracts and full-text articles, extracted the data, and performed statistical analysis. VB and JK designed the study, analyzed, interpreted the data, drafted, and finalized the manuscript. JK, DE, and AM conceived the original research idea, participated in data interpretation, theoretical interpretation, and description, and gave input on report drafts. All authors contributed to the article and approved the submitted version.

## Conflict of Interest

DE was President and Chief Executive Officer of Canadian Health Solutions Inc. Canadian Health Solutions is currently researching potential clinical indications correlated with a diagnosis of PTSD. The current research has no direct commercial relationship with that work. AM was employed by Canadian Health Solutions Inc. The remaining authors declare that the research was conducted in the absence of any commercial or financial relationships that could be construed as a potential conflict of interest.

## Publisher’s Note

All claims expressed in this article are solely those of the authors and do not necessarily represent those of their affiliated organizations, or those of the publisher, the editors and the reviewers. Any product that may be evaluated in this article, or claim that may be made by its manufacturer, is not guaranteed or endorsed by the publisher.
