# Supplementary material for: A Systematic Review and Meta-Analysis of Lipid Signatures in Post-traumatic Stress Disorder
Source: Front Psychiatry. 2022 May 6;13:847310. doi: 10.3389/fpsyt.2022.847310 (PMC9120430; doi:10.3389/fpsyt.2022.847310)
Supplement: Supplementary file 1 [file Table_1.DOCX]

***Supplemental Information***

**A Systematic Review and Meta-Analysis of Lipid Signatures in Post-Traumatic Stress Disorder**

**Veni Bharti, PhD^1,2^, Aseem Bhardwaj, MSc^2^, David A. Elias, MD,** **CCFP(EM)(AM), CIME, FCBOM ^3,4^, Arron W.S. Metcalfe, PhD^3,5^, Jong Sung Kim, PhD*^1,2^**

^1^ Department of Community Health and Epidemiology, Faculty of Medicine, Dalhousie University, Canada

^2^ Health and Environments Research Centre (HERC) Laboratory, Faculty of Medicine, Dalhousie University, Canada

^3^ Canadian Health Solutions, Canada

^4^ Dalhousie Medicine New Brunswick, Dalhousie University, Canada

^5^ Canadian Imaging Research Centre, Canada

***Correspondence:**

Dr. Jong Sung Kim

[jskim@dal.ca](mailto:jskim@dal.ca)

***Search Strategy***

1. ‘cholesterol’/exp
2. Cholesterol: ti,ab,kw
3. ‘lipoprotein’/exp
4. Lipoprotein* : ti, ab,kw
5. ‘high density lipoprotein’/exp
6. ‘high density lipoprotein’: ti,ab,kw
7. hdl: ti,ab,kw
8. ‘high density lipoprotein cholesterol’/exp
9. ‘low density lipoprotein’/exp
10. ‘low density lipoprotein’: ti,ab,kw
11. ldl: ti,ab,kw
12. ‘low density lipoprotein cholesterol’/exp
13. ‘very low density lipoprotein’/exp
14. ‘very low density lipoprotein’: ti,ab,kw
15. vldl: ti,ab,kw
16. ‘very low density lipoprotein’/exp
17. Triglyceride*:ti,ab,kw
18. Tg:ti,ab,kw
19. ‘hyperlipidemia’/exp
20. Hyperlip*:ti,ab,kw
21. ‘hypobetalipoproteinemia’/exp
22. Hypobetalipoproteinemia: ti,ab,kw
23. Hypolip*: ti,ab,kw
24. ‘hypercholesterolemia’/exp
25. Hypercholesterolemia: ti,ab,kw
26. ‘posttraumatic stress disorder’/exp
27. ‘posttraumatic stress disorder’:ti, ab,kw
28. Ptsd: ti,ab,kw
29. ‘post-traumatic stress disorder’: ti,ab,kw
30. #1 OR #2 OR #3 OR #4 OR #5 OR #6 OR #7 OR #8 OR #9 OR #10 OR #11 OR #12 OR #13 OR #14 OR #15 OR #16 OR #17 OR #18 OR #19 OR #20 OR #21 OR #22 OR #23 OR #24 OR #25
31. #26 OR #27 OR #28 OR #29
32. #30 AND #31
